# Evidence based recommendations for genetic diagnosis of Familial Mediterranean Fever

**DOI:** 10.1186/1546-0096-12-S1-P241

**Published:** 2014-09-17

**Authors:** Gabriella Giancane, Nienke Ter Haar, Nico Wulffraat, Bas Vastert, Karyl Barron, Veronique Hentgen, Kallinich Tilmann, Huri Ozdogan, Jordi Anton Lopez, Paul Brogan, Luca Cantarini, Joost Frenkel, Caroline Galeotti, Marco Gattorno, Gilles Grateau, Michael Hofer, Isabelle Kone-Paut, Jasmin Kuemmerle-Deschner, Helen Lachmann, Anna Simon, Brian Feldman, Yosef Uziel, Seza Ozen

**Affiliations:** 1 Department of Pediatric Immunology, UMC, Utrecht, Netherlands; 2 NIH, Bethesda, USA; 3 Centre Hospitalier de Versailles, Le Chesnay Cedex, France; 4 Charite University Medicine, Berlin, Germany; 5 Cerrahpasa Ic Hastaliklari Klinigi, Istanbul, Turkey; 6 Hospital Sant Joan de Déu, Universitat de Barcelona, Barcelona, Spain; 7 Department of Rheumatology, UCL Institute of Child Health, London, UK; 8 Policlinico Le Scotte, University of Siena, Siena, Italy; 9 Algemene Pediatrie, UMC, Netherlands; 10 Reference Centre for Autoinflammatory Disorders CEREMAI, Bicêtre Hospital, University of Paris SUD, Le Kremlin Bicêtre Cedex, France; 11 G. Gaslini Institute, Genova, Italy; 12 Hôpital Tenon,AP-HP, université Pierre-et-Marie-Curie, Centre National de Référence des Amyloses d'Origine Inflammatoire et de la Fièvre, Paris, France; 13 Department of Paediatrics, University of Lausanne, Lausanne and University of Geneva, Geneva, Switzerland; 14 Division of Paediatric Rheumatology, Reference Centre for Autoinflammatory Disorders CEREMAI, Bicêtre Hospital, University of Paris SUD, Paris, France; 15 Klinik für Kinder- und Jugendmedizin, Abteilung für pädiatrische Rheumatologie, Autoinflammation Reference Center Tübingen, Universitätsklinikum Tübingen, Tübingen, Germany; 16 National Amyloidosis Centre, University College London Medical School, London, UK; 17 Department of Medicine, Division of General Internal Medicine, Radboud University Nijmegen Medical Center, Nijmegen , Netherlands; 18 University of Toronto, The Hospital for Sick Children, Toronto, Canada; 19 Department of Pediatrics, Sapir Medical Center, Kfar Saba, Tel-Aviv University, Sackler School of Medicine, Tel-Aviv, Israel; 20 Department of Pediatrics, Hacettepe University Faculty of Medicine, Ankara, Turkey

## Introduction

Familial Mediterranean Fever (FMF) is a disease that starts in childhood and can lead to significant morbidity. In 2012, a European initiative called SHARE (Single Hub and Access point for pediatric Rheumatology in Europe) has been launched to optimize and disseminate diagnostic and management regimens in Europe for children and young adults with rheumatic diseases. For FMF, attention was focused on genetics.

## Objectives

The aim of the SHARE recommendations in FMF is to provide a diagnostic tool for inexperienced pediatric rheumatologists to cope with FMF in their clinical practice. This is possible through a correct interpretation of the diagnostic value of *MEFV* gene mutations in predicting FMF phenotype.

## Methods

Evidence-based recommendations were developed using the European League Against Rheumatism (EULAR) standard operating procedure. An expert committee was instituted, consisting of pediatric rheumatologists, and search terms for the systematic literature review were defined. Two independent experts scored articles for validity and level of evidence. Recommendations derived from the literature were evaluated by an online survey. Those with less than 80% agreement during the online survey were reformulated. Subsequently, all recommendations were discussed at a consensus meeting using the nominal group technique. Recommendations were accepted if more than 80% agreement was reached.

## Results

The literature search yielded 3386 articles, of which 240 about genetics and, among them, 25 considered relevant and therefore scored for validity and level of evidence. 17 articles were scored valid and used in the formulation of the recommendations. 9 recommendations for diagnosis were suggested in the online survey and 8 were finally accepted with 100% agreement after the consensus meeting. Topics covered for diagnosis were: clinical versus genetic diagnosis of FMF; genotype – phenotype correlation; genotype – age at onset correlation; silent carriers and risk for amyloidosis; role of the specialist in FMF diagnosis.

## Conclusion

The SHARE initiative provides recommendations for the diagnosis of FMF and thereby facilitates improvement and uniformity of care throughout Europe.

## Disclosure of interest

G Giancane: None declared, N Ter Haar Grant / Research Support from: SHARE is funded by the European Commission ( project N° 20111202), N. Wulffraat Grant / Research Support from: Abbvie, GSK, Roche, Consultant for: Genzyme, Novartis, Pfizer, Roche, B. Vastert Consultant for: Novartis, K Barron: None declared, V Hentgen: None declared, K Tilmann Grant / Research Support from: Novartis, Speaker Bureau of: Novartis, SOBI, H Ozdogan: None declared, J Anton Lopez Grant / Research Support from: Abbvie, Novartis, Pfizer , Consultant for: Novartis, Speaker Bureau of: Abbvie, Novartis, Pfizer, Roche, SOBI, P Brogan Grant / Research Support from: Novartis, Roche, Consultant for: Roche, L Cantarini: None declared, J Frenkel Consultant for: Novartis, Speaker Bureau of: SOBI, C Galeotti Grant / Research Support from: Novartis, M Gattorno: None declared, G Grateau: None declared, M Hofer: None declared, I Kone-Paut: None declared, J Kuemmerle-Deschner Grant / Research Support from: Novartis, Speaker Bureau of: SOBI, H Lachmann: None declared, A Simon Consultant for: Novartis, Xoma, SOBI, B Feldman: None declared, Y Uziel Grant / Research Support from: Novartis , Consultant for: Novartis, Speaker Bureau of: Abbvie, Neopharm, Novartis, Roche, S. Ozen Grant / Research Support from: Novartis , Speaker Bureau of: Biovitrium

